# Does Technical Match Performance in Professional Soccer Depend on the Positional Role or the Individuality of the Player?

**DOI:** 10.3389/fpsyg.2022.813206

**Published:** 2022-05-31

**Authors:** Leon Forcher, Leander Forcher, Sascha Härtel, Darko Jekauc, Hagen Wäsche, Alexander Woll, Timo Gross, Stefan Altmann

**Affiliations:** ^1^Institute of Sports and Sports Sciences, Karlsruhe Institute of Technology, Karlsruhe, Germany; ^2^TSG 1899 Hoffenheim, Zuzenhausen, Germany; ^3^TSG ResearchLab gGmbH, Zuzenhausen, Germany

**Keywords:** team sports, football, TACTICS, passing, dribbling, technical performance

## Abstract

The aim of the study was to examine the impact of the positional role and the individuality on the technical match performance in professional soccer players. From official match data of the Bundesliga season 2018/19, technical performance [short (<10 m)/medium (10–30 m)/long (>30 m) passes, dribblings, ball possessions] of all players who played during the season were analyzed (normative data). Five playing positions (center back, full back, central midfielder, wide midfielder and forward) were distinguished. As the contextual factor tactical formation is known to influence match performance, this parameter was controlled for. Further, those players who played at minimum four games in at least two different playing positions were included in the study sample (*n* = 13). The technical match performance of the players was analyzed in relation to the normative data regarding the extent to which the players either adapted or maintained their performance when changing the playing position. When switching playing positions, positional role could explain 3–6% of the variance in short passes and ball possessions and 27–44% of the variance in dribblings, medium passes, and long passes. Moreover, we observed large interindividual differences in the extent to which a player changed, adapted, or maintained his performance. In detail, five players clearly adapted their technical performance when changing playing positions, while five players maintained their performance. Coaches can use these findings to better understand the technical match performance of single players and further, to estimate the impact of a change in the positional role on the technical performance of the respective player.

## Introduction

Soccer match performance is determined by a complex interaction of numerous factors including tactical, physical, and technical aspects (Sarmento et al., 2014). In this context, tactical factors like the playing position and the tactical formation have been shown to affect the physical as well as the technical match performance of professional soccer players (Bradley et al., 2011; Dolci et al., 2020).

In terms of physical performance, wide playing positions (e.g., full backs and wide midfielders) generally cover greater distances at high-intensity and sprinting speed than central positions (e.g., center backs; Dellal et al., 2010; Rivilla-Garcia et al., 2018; Aquino et al., 2020). Further, the technical performance of both wide and central midfielders is characterized by a higher number of ball possessions compared to other positions (Dellal et al., 2010). Relating to the tactical formation players in a 3-5-2 formation cover more total distance (Bradley et al., 2011; Palucci Vieira et al., 2018; Aquino et al., 2019) and players in a 4-2-3-1 formation accelerate more often (Tierney et al., 2016; Arjol-Serrano et al., 2021) than in other tactical formations. Moreover, players in a 4-4-2 formation play more passes than players in other formations (Bradley et al., 2011; Arjol-Serrano et al., 2021).

In addition to investigating the isolated effect of playing position and tactical formation, combining both tactical factors provide deeper insights into how tactical aspects influence physical and technical match performance. This combination of tactical factors playing position (e.g., center back) and tactical formation (e.g., 4-4-2) will be defined as *positional role*. Specifically, a player’s positional role consists of 1. the playing position and 2. the tactical formation leading to a combined phrase like’ *center back in 4-4-2′*. Using the combination of both tactical factors results in more detailed outcomes. Further, the results throughout the studies become more consistent. For example, previous studies revealed that the physical match performance of central defenders, wide defenders, and attackers is higher in a 3-5-2 formation than in a 4-4-2 formation (Tierney et al., 2016; Baptista et al., 2019; Borghi et al., 2020; Modric et al., 2020; Arjol-Serrano et al., 2021). An example that illustrates this connection once again is that center backs cover the greatest sprinting distance in a 3-5-2 formation (Baptista et al., 2019; Modric et al., 2020) and the least sprinting distance in a 4-4-2 formation (Modric et al., 2020; Arjol-Serrano et al., 2021).

Nevertheless, the match performance of soccer players is not only dependent on a positional role (i.e., combination of playing position and tactical formation) but also depends on various contextual factors. Examples for those factors include the league and country being played in, the opponent strength, or whether the match is at home or away (Rampinini et al., 2007; Dellal et al., 2011; Trewin et al., 2017; Aquino et al., 2020). Another factor that influences the match performance is the individuality of the respective player. An interesting observation of practitioners is that certain players always show similar match performances even if they play in different positional roles. A logical conclusion of this practical observation could be that soccer match performance is less dependent of the positional role and more strongly associated with the individual player. Each player has his unique set of skills and abilities. These individual factors influence match performance and therefore, can help to explain interindividual differences in single player match performances.

Two studies already examined the extent to which the match performance of soccer players is not only position-specific, but also player-specific (Schuth et al., 2016; Altmann et al., 2021). Altmann et al. (2021) investigated the behavior of players that switched between playing positions (e.g., center back to full back). Their results showed that 44–58% of the intraindividual changes in physical match performance due to the change in position can be explained by the factor playing position. Another interesting finding was that for players that switched from center back to full back, a higher physical performance was observed when these players acted as a full back (vs. playing as a center back). Further, this result follows normative data which also indicate a higher physical performance for full backs in comparison to center backs. This finding is also observed in the investigation of Schuth et al. (2016). Further, both studies observed high interindividual differences in the way players either adapted or maintained their physical match performance when changing their playing position.

As already mentioned, different players possess different technical and physical skills and might also interpret their playing position differently. This can lead to different technical or physical match performances of individual players, even though they play the same position. Nevertheless, these two studies focused only on the tactical aspect of playing position and did not consider the tactical formation as an additional factor affecting physical or technical match performance. Further, Altmann et al. only focused on physical aspects of match performance. Hence, it is worth investigating the technical soccer match performance on this topic, considering the tactical formation as well. In the present paper, the tactical formation (e.g., 4-4-2) will be controlled as a combined factor with the playing position (e.g., center back), resulting in different positional roles (e.g., *‘center back in 4-4-2’*).

Therefore, the aim of this study was to examine to what extent the technical match performance of professional soccer players is dependent on the positional role or on the individuality of the respective player. To address this question, we evaluated data of players switching positional roles and normative positional data in relation to each other. The normative data consists of all players that participated in the study period. We used an idiographic study design to analyze the behavior of individual players. In contrast to a nomothetic approach, an idiographic approach investigates individual cases to describe and interpret them in the respective context. Based on the results of Altmann et al. (2021) we hypothesize that some players will maintain their performance while some players will adjust their performance toward the positional role.

## Materials and Methods

### Study Design

In this study, official match data from the 2018/2019 season of the German Bundesliga were used, since this was the last season that has not been affected by the COVID-19 pandemic (Thron et al., 2021). A total of 267 games were analyzed, as every match with at least one sent-off (39 games) was excluded. Only players that were involved in the whole respective game (i.e., full 90 min) were included, leading to a maximum of 20 outfield players per match. The 474 players that participated in this season lead to 3,810 single players match performances that were analyzed in this study (normative data).

First, the tactical formation of each team and the playing position of each player who took part in at least one of the included 267 matches of this season were recorded. Five different playing positions (center back, full back, central midfielder, wide midfielder, forward) and eight tactical formations were distinguished (4-4-2, 4-4-2 diamond, 4-2-2-2, 4-3-3, 4-5-1, 4-2-3-1, 3-4-3, 3-5-2).

To determine whether technical performance is not only specific to the positional role but also to the individual athlete, players that played in at least two different positional roles [(i.e., playing position = e.g., center back) and (tactical formation = e.g., 4-4-2): positional role = ‘*center back in 4-4-2’*] were identified. Therefore, the technical performance for the players’ first and second positional role was examined independently. Further, the possible differences in technical performance that occurred when players switched their playing position was analyzed. For all players that were included, the technical performance was analyzed [dribblings, short passes (<10 m), medium passes (10–30 m), long passes (>30 m), ball possessions]. Data were collected as part of the players’ professional employment so that ethical approval was not required for this study (Winter and Maughan, 2009).

### Subjects

To be included in the study sample, players must have completed at least four entire matches (i.e., 90 min) in at least two different positional roles (i.e., one playing position in a respective tactical formation: e.g., 4 games: ‘*center back in 4-4-2’*, 4 games: ‘*full back in 4-3-3’*). Consistent with Altmann et al. (2021), only players that changed their playing position were included in the study sample. Therefore, players that combined two positional roles (e.g., *‘center back in 4-4-2’* and *‘center back in 4-3-3’*) representing only one playing position (e.g., center back) were excluded. On the other hand, if a player played on two positional roles (e.g., *‘center back in 4-4-2’* and *‘full back in 4-4-2’*) while the formation (e.g., 4-4-2) did not change, he was included in the sample. A minimum of four games per positional role was used to account for the variability of technical performance between matches and to minimize the influence of contextual factors (Bradley et al., 2011; Aquino et al., 2019; Arjol-Serrano et al., 2021). As a result, 13 players were included in the study sample.

Normative data for every positional role were collected simultaneously. The normative data consisted of 3,810 single-player match performances (i.e., each single-player match performance of all players that played at least one entire match of the 267 games that were analyzed). These normative data provide information about the typical technical match performance of players representing the specific positional role in the German Bundesliga season 2018/19.

### Procedures

Every player (study sample and normative data) was assigned to a positional role representing the playing position in a corresponding tactical formation (see Supplementary Table 1). The tactical formations are constructed out of the starting eleven and reviewed by observation after 15 min of each match. To analyze the accuracy of the provided tactical formation data, we validated the formations provided for the first match day of the 18/19 season (9 games, 18 formations) by the observation of an experienced game and video analyst of the German Bundesliga team TSG 1899 Hoffenheim. Given the high agreement between the results of the provided formations and the observations of the video analyst (Cohen’s Kappa: 0.93, *p* < 0.05), the data provided by Deltatre (Deltatre, Turin, Italy) were used in this study (Landis and Koch, 1977). For each player in the study sample, the first and the second positional role was determined. If a player combined two positional roles (e.g., *‘center back in 4-4-2’* and *‘center back in 4-3-3’*) representing only one playing position (e.g., center back), the subject was excluded. If a player reached the minimum number of four matches for three different positional roles, the positional role with the least games was excluded (e.g., Player 1: four games: ‘*center back in 4-4-2’*, five games: ‘*center back in 4-3-3’*, eight games: ‘*full back in 3-4-3’*; - > only ‘*center back in 4-3-3’* and ‘*full back in 3-4-3’* were analyzed). Therefore, the players in the study sample combined two positional roles, with both positional roles representing two different playing positions. This ensures comparability of the results with previous studies (Schuth et al., 2016; Altmann et al., 2021). We used an idiographic study design. Therefore, the variability of performance changes that cannot be attributed to the positional role could potentially be associated with the individuality of the respective player.

The technical performance was analyzed using the number of dribblings, passes, and ball possessions. Throughout previous studies that investigated technical match performance, ball possessions, passes and dribblings were the most frequently analyzed parameters (Bradley et al., 2011; Aquino et al., 2019; Arjol-Serrano et al., 2021). Based on the covered distance of the ball, passes were divided into three categories [short (<10 m), medium (10 ≥ 30 m), long (>30 m)]. One dribbling was counted if one player attempted to dribble past an opponent while safely in control of the ball. One ball-possession phase for one player was counted when he had a ball action in a ball-possession phase of his team. All definitions are based on the catalog of the German soccer league (Deutsche Fußball Liga, 2019).

The technical performance was conducted using the DFL Observed Tracking-Data processed by Deltatre. The data are based on a Multi-Camera-Tracking System (TRACAB, Chyron Hego, Melville, NY, United States), that was previously validated (Linke et al., 2020).

### Statistical Analysis

All statistical analyzes were executed using IBM SPSS Statistics 25.0.0.0 (IBM Co., New York, United States). The significance level for all tests was set to 0.05. Mean values and standard deviations (SD) were calculated for each player of the study sample and for the normative data for each playing position in the different tactical formations.

To determine possible performance changes when changing the positional role, the study sample data and the normative data were analyzed in relation to each other. The differences between the technical performance between the first and the second positional role of player of the study sample were tested by independent *t*-tests and represented by Cohen’s d effect sizes (ES). Small (0.2 ≤ ES < 0.5), medium (0.5 ≤ ES < 0.8) and large (ES ≥ 0.8) ES were distinguished (Cohen, 1988). Further, Pearson’s product–moment correlations with 95% confidence intervals (95% CI) were calculated between the positional difference of the players in the study sample and the associated positional difference in the normative data. This correlation helps to quantify the contribution of the positional role in the variability between the first and second positional role of the players in the sample. The correlation coefficients were classified into small (0.1 ≤ *r* ≤ 0.3), moderate (0.3 ≤ *r* ≤ 0.5), large (0.5 ≤ *r* ≤ 0.7), very large (0.7 ≤ *r* < 0.9), and nearly perfect (*r* ≥ 0.9; Hopkins, 2002).

## Results

Regarding the study sample, six players (players 6, 7, 8, 9, 10 and 11) combined the playing positions of wide midfielder and central midfielder, while their positional roles were different (i.e., different tactical formations). Moreover, two players combined the playing positions center back and central midfielder (players 1 and 2), and wide midfielder and forward (players 12 and 13), representing different positional roles, respectively. The other playing position combinations [center back/ full back (player 3); wide midfielder/full back (player 4); central midfielder/forward (player 5)] were represented by one player, each.

For the technical parameters short passes and ball possessions, the correlation between the positional performance difference of the players in the study sample and the respective normative data was small (r-range = 0.18–0.24; *r*^2^-range = 3–6%; See Table 1). For the parameters dribblings, medium passes, and long passes this correlation was large (r-range = 0.52–0.66; *r*^2^-range = 27–44%).

**Table 1:** Pearson’s r (*r*^2^), 95% CI and values of *p* for correlations between the positional difference of the players in the study sample and the associated positional difference in the normative data for dribblings, short passes, medium passes, long passes, and ball possessions.

	Dribblings	Short passes	Medium passes	Long passes	Ball possessions
Person’s r (*r*^2^)	0.66 (44%)	0.18 (3%)	0.54 (29%)	0.52 (27%)	0.24 (6%)
95% CI	0.18–0.89	−0.42 to 0.66	−0.02 to 0.84	−0.05 to 0.83	−0.36 to 0.70
Value of *p*	0.01	0.57	0.06	0.07	0.44

**Figure 1:**
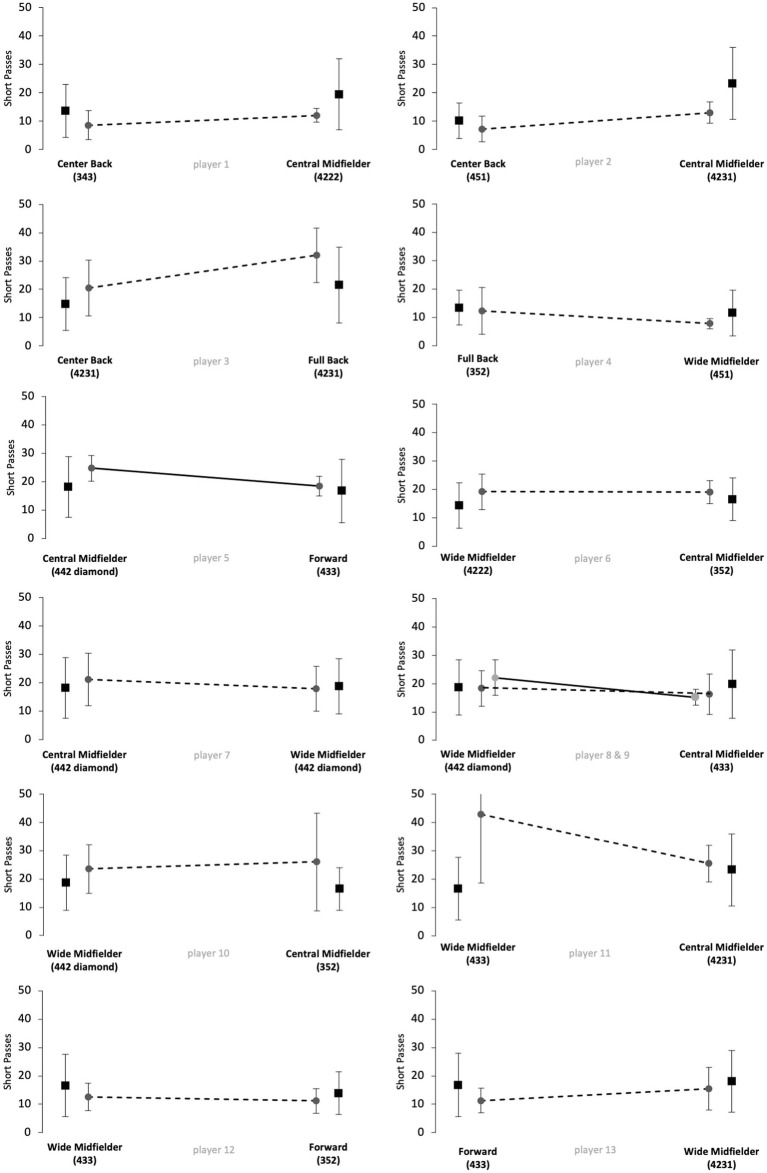
Number of short passes of players from the study sample (grey circles) in relation to normative positional data (black squares). Data are presented as means ± SD for the respective games played on the respective positional role. Solid lines indicate significant differences in performance between the two positions for the respective player.

**Figure 2:**
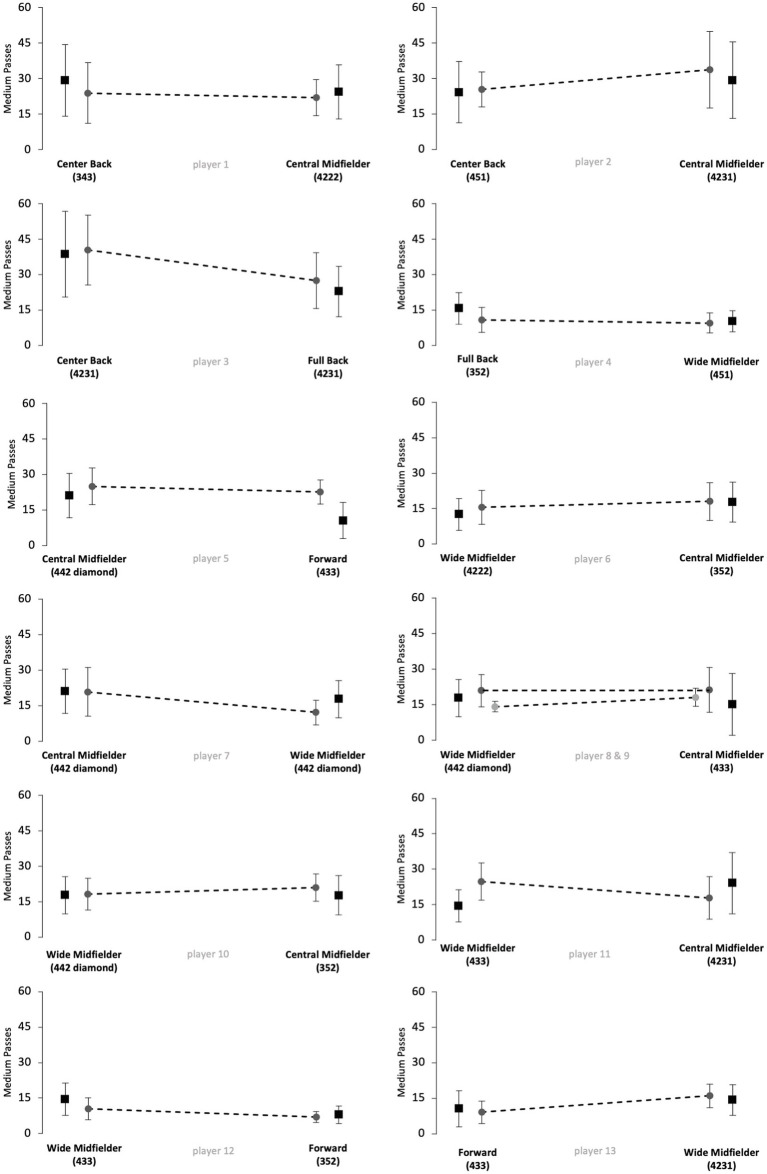
Number of medium passes of players from the study sample (grey circles) in relation to normative positional data (black squares). Data are presented as means ± SD for the respective games played on the respective positional role. Solid lines indicate significant differences in performance between the two positions for the respective player.

**Figure 3:**
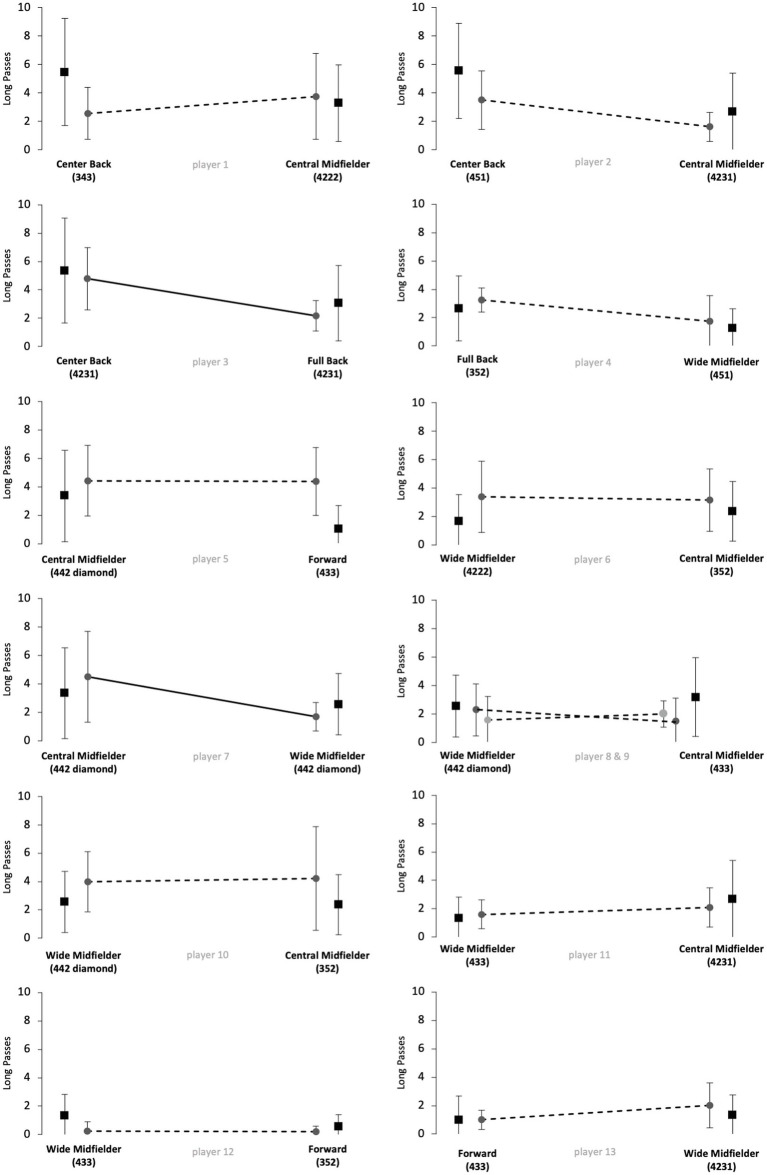
Number of long passes of players from the study sample (grey circles) in relation to normative positional data (black squares). Data are presented as means ± SD for the respective games played on the respective positional role. Solid lines indicate significant differences in performance between the two positions for the respective player.

**Figure 4:**
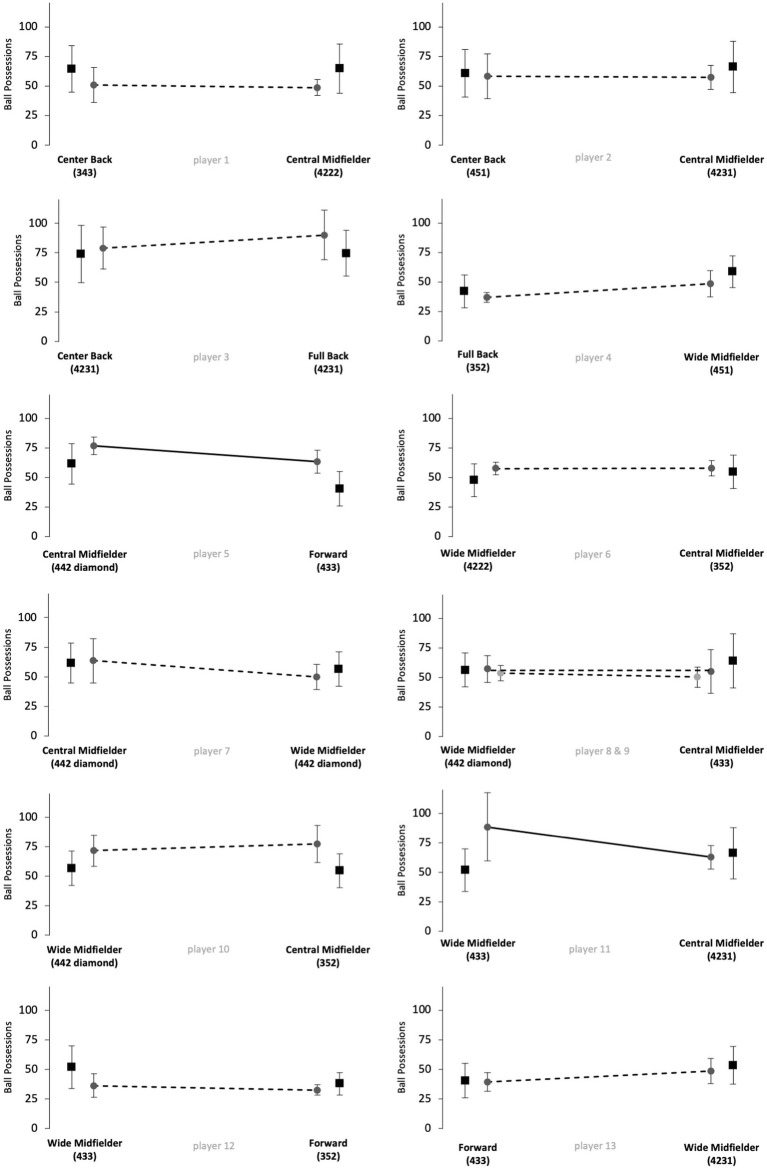
Number of ball possessions of players from the study sample (grey circles) in relation to normative positional data (black squares). Data are presented as means ± SD for the respective games played on the respective positional role. Solid lines indicate significant differences in performance between the two positions for the respective player.

**Figure 5:**
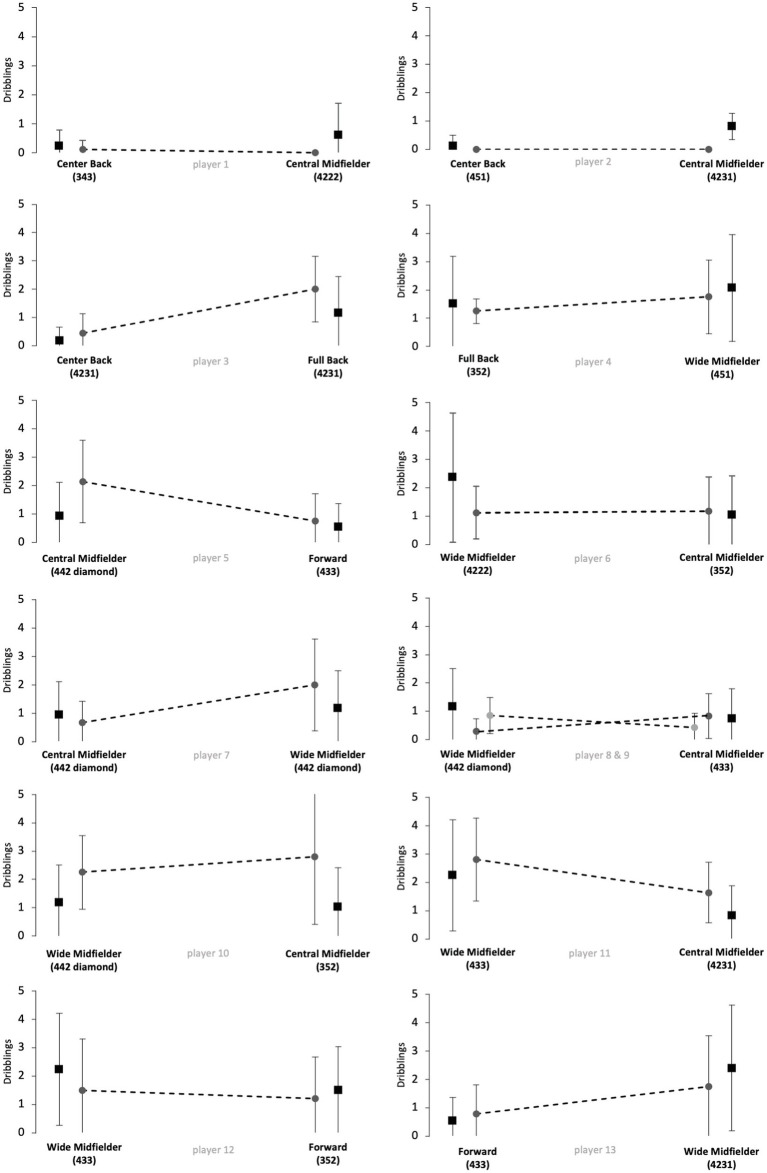
Number of dribblings of players from the study sample (grey circles) in relation to normative positional data (black squares). Data are presented as means ± SD for the respective games played on the respective positional role. Solid lines indicate significant differences in performance between the two positions for the respective player.

Figures 1–5 show the technical performance of the players of the study sample in relation to the respective normative data of the positional role. Descriptive values (means ± SD), *t*-test results, and ES regarding the study sample are presented in Supplementary Table 2.

Five players (players 1, 6, 8, 10 and 12) rather maintained their technical performance when changing the positional role, as indicated by a maximum of two large ES in the five reported parameters. However, five players (players 3, 5, 7, 11 and 13) apparently changed their technical performance when changing the positional role, as indicated by at least four large ES in the reported performance parameters. The other 3 players (players 2, 4 and 9) showed large ES in three of the five parameters and therefore revealed inconsistent differences when changing the positional role. Further, we observed large interindividual differences in the way players adapted or maintained their performance when changing the playing position. Players showed different magnitudes in performance changes and these differences individually occurred in different performance parameters.

Additional descriptive information about the players of the study sample can be found in Supplementary Table 7. Eight players were born in Germany, while player 3 (Senegal), player 6 (Austria), player 7 (Ivory Coast), player 9 (Netherlands), and player 12 (France) were born in other countries. Except for players 1, 2, and 7 all players belong to teams that finished the season in the top half of the table. Furthermore, four players (players 3, 6, 10, and 11) were active in international competition during the study period. Moreover, the players of the study sample played in 85% of all league matches during the study period.

Moreover, the normative data reveals further information. Regarding playing positions (see Supplementary Table 4) the most obvious results were that center backs and full backs reveal more ball possessions compared to the other playing positions. Furthermore, center backs play the most short and medium passes of all playing positions. Moreover, combining the playing position and formation reveals deeper insights (see Supplementary Table 3). While center backs, full backs, and wide midfielders reveal larger differences between formations, the technical performance of central midfielders and forwards differed only slightly between the formations.

## Discussion

The aim of the current study was to examine to what extent the technical match performance of professional soccer players is dependent on the positional role (i.e., a combination of playing position and tactical formation) or on the individuality of the respective player. Positional role could explain 3–6% of the variability in short passing and ball possessions and 27–44% of the variability in dribbling, medium passing, and long passing. The remaining variability in the respective parameters can be attributed to different influencing factors including the individuality of each player. The results showed large differences in the way the players adapted or maintained their technical match performance when changing positional roles.

The results of the normative data on technical match performance revealed conflicting outcomes to previous investigations. Center backs and full backs seemed to display the most ball possessions compared to other playing positions. Previous investigations found that central and wide midfielders had the most ball possessions (Dellal et al., 2010). While in the past midfielders were the playmakers, in recent years defensive positions (e.g., center back) have been given more and more responsibility in shaping the game. For example, Bush et al. (2015) found that the number of passes from center backs has increased in the last decade. Importantly this increase was larger for center backs than for the remaining playing positions (Bush et al., 2015). Therefore, the conflicting results could potentially be associated with the data by Dellal et al. (2010) being collected over a decade ago. Further, the center backs played the most medium and long passes, while together with forwards playing the fewest short passes. Central midfielders played the most short passes and wide midfielders displayed the most dribblings of all positional groups. Moreover, the results indicated that the tactical formation has an effect on technical match performance (see Supplementary Table 5). Further, the influence of tactical formation on technical performance is position-dependent (see Supplementary Table 3). While the technical performance of center backs, full backs, and wide midfielders differed markedly between tactical formations, central midfielders and forwards showed smaller differences between the tactical formations. These results indicate that the tactical formation needs to be considered when looking at the match performance of soccer players.

To figure out how players adapt or maintain their technical match performance when changing the positional role, we analyzed the results of the study sample in relation to the normative data for each positional role. The correlation between the positional performance difference of the players in the study sample and the respective differences in the normative data revealed differences depending on the parameter. For the parameters short passes and ball possessions, the respective positional roles could explain only 3–6% of the variability. Regarding the parameters dribblings, medium passes, and long passes the positional roles explained 27–44% of the variability. Therefore, short passes and ball possessions underlie less influence of the positional role, while the influence of the positional role on medium passes, long passes, and dribblings is markedly larger. This finding could be associated with the heterogenous normative positional data. Larger variability in the normative data promoted higher correlations. While the normative positional data show large differences between the playing positions regarding the parameters medium passes, long passes, and dribblings, the differences regarding short passes and ball possessions are much smaller. For example, wide midfielders show 980% more dribblings than center backs and center backs reveal 777% more long passes than forwards. Therefore, the results of the correlation regarding these parameters can strongly be linked with the normative data regarding the playing positions. The results of Altmann et al. (2021), who used a similar strategy in study design and methods, showed that physical performance was influenced by position to a greater extent than technical performance. These results can indicate that the influence of the playing position on technical performance is smaller than on physical performance. Therefore, we could potentially conclude that the individual playing style of the respective player has a larger impact on technical performance than on physical performance.

The results of Figures 1–5 and Supplementary Table 2 indicate large interindividual differences in adaption or maintenance of the technical match performance when changing the positional role. Regarding their reaction to switching positional roles, the players in the study sample could be categorized into three different groups.

The first group consists of five players that markedly changed their technical performance when changing the positional role, indicated by at least four large ES in the five analyzed parameters (players 3, 5, 7, 11 and 13). Two players represented the position combination wide midfielder and central midfielder, while the other position combinations (center back/full back; central midfielder/forward; wide midfielder/forward) were only represented by one player.

The second group is represented by five players who tended to maintain their technical performance when changing the positional role, as indicated by a minimum of two large ES in the five technical parameters (players 1, 6, 8, 10 and 12). Three of those players changed between the positions of wide midfielder and central midfielder. The position combinations center back and central midfielder, as well as wide midfielder and forward, were represented only once.

The remaining three players indicated three large ES in the examined parameters and therefore revealed inconsistent changes when changing the positional role (players 2, 4 and 9). Each position combination was represented by one player (center back/central midfielder; full back/wide midfielder; wide midfielder/central midfielder).

The way single players changed or maintained their technical performance when changing the positional role highlights large interindividual differences. For instance, players 12 and 13 represented the same position combination (wide midfielder/forward) but behaved markedly different when changing from wide midfielder to forward. In detail, player 12 rather maintained his technical performance for the parameters dribblings, short passes, long passes, and ball possessions and only adapted his performance towards the normative positional data regarding medium passes (decrease from wide midfielder to forward). In contrast, player 13 adapted his performance toward the normative positional data in all five technical performance parameters. In detail, player 13 revealed a decreasing number of passes (short, medium, and long), ball possessions, and dribblings when switching from wide midfielder to forward. Both players played in 100% games of their teams in the investigated Bundesliga season and finished the season in a top table position, respectively, (i.e., table position 4 and 5, see Supplementary Table 7). However, the team of player 13 was active in the Europa League throughout the study period. The additional number of games could mean that the match performance of player 13 was affected by the enormous number of games (Folgado et al., 2015; Palucci Vieira et al., 2018). Overall, the differences that occur when players change positional roles are multivariate. Accordingly, the change in the playing position as well as the change in the tactical formation can affect performance. In contrast to the results of Altmann et al. (2021), who used a similar study design (e.g., Bundesliga) and had different explanation approaches for the performance changes of individual players in the study sample, the results of the current study were more heterogeneous (Altmann et al., 2021). Logical explanations as why single players in the current study sample adapted or maintained their technical performance in the respective parameters could not be derived.

In the study of Schuth et al. (2016), the technical performance of players who changed playing positions from center back to full back changed according to the normative data for each position (Schuth et al., 2016). The only player representing this positional interchange combination in the present study (player 3) revealed a similar reaction in adapting his technical performance. Moreover, Schuth et al. (2016) revealed that players changing from central midfielder to wide midfielder showed fewer passes and ball possessions in their second position, which also followed the normative positional data. In our study, six players represent the mentioned position combination (i.e., center back/full back). Two players (players 7 and 11) adapted their technical performance toward the normative positional data. The other three players (players 6, 8 and 10) tended to maintain their performance and, therefore, were somehow unaffected by the change of positional roles. One player (player 9) showed an alternating behavior when switching from central to wide midfielder and, therefore, could not be assigned to either group. One possible explanation for the conflicting results could be that Schuth et al. (2016) examined data of seven consecutive seasons. Single players develop during this long period of time and might also adapt to the evolving game, which became more technically demanding during this time span (Bush et al., 2015). Further, Schuth et al. (2016) did not consider tactical formations, which might limit the generalizability of their results.

The players of the sample were regulars who played in 85% of all league matches (see Supplementary Table 7). With the exception of players 3 (Senegal), 6 (Austria), 7 (Ivory Coast), 9 (Netherlands), and 12 (France), all players were born in Germany. Most of the players in the sample played for clubs that finished the season in the top half of the table. Only players 1, 2, and 7 were not as successful with their respective teams. However, all players managed to prevent their teams from relegation. Only four players (players 3, 6, 10, and 11) were active with their teams in the Champions League or the Europa League.

Moreover, the results can be discussed from an ecological dynamics perspective. Previous studies revealed that a players’ main playing position (e.g., defender, midfielder, or forward) has a significant influence on the technical-tactical elements in soccer (Laakso et al., 2019, 2021). Therefore, from an ecological dynamics perspective, when considering the results, it must be taken into account that the main player position has an influence on the perception-action systems of soccer players (Araújo et al., 2006). Since the players mostly play in their main playing position during their development in soccer, they learn to perceive, process and implement the position-specific technical-tactical elements of the soccer game. From these points of view, the players from the current study sample need to be considered specifically. To be more precise, the players in this sample are regulars at different main positional roles and, therefore, cannot be assigned to a single specific main playing position. From an ecological dynamics perspective, it would be profitable to test the findings of Laakso et al. (2019, 2021) using such special player samples. The results from such studies might indicate in what way these theories also apply to this particular type of player.

The technical performance of soccer players varies from match to match (Bush et al., 2015). Therefore, we tried to minimize the effect of single match performances by extending the criterion for inclusion in the study sample to a minimum of four games per positional role. Because we considered the tactical formation and playing position for each player, this simultaneously led to a small sample size (*n* = 13), which can be considered a limitation of this study. However, these strict inclusion criteria lead to more meaningful outcomes compared to a larger sample size that would result from a smaller number of games as an inclusion criterion. In the study sample, 12 out of 13 players played as midfielders (central or wide) in at least one of the two positional roles considered per player. Therefore, midfielders represented a majority of the study sample. This finding could be associated with the assumption that midfielders are more flexible in terms of positional roles than players in other playing positions. More specifically, midfielders are strongly integrated into both the attack and defense, while the task focus of forwards and defenders is either attack or defense. Hence, midfielders could possibly better fill a second defensive (center back or full back) or offensive position (forward) in addition to their main midfield position (central or wide). Moreover, we only analyzed five different technical performance parameters. To provide a full picture of the technical performance of a professional soccer player more different technical performance parameter would be desirable in future studies. Another limitation of this study is that contextual factors are only provided for the tactical formations (see Supplementary Table 6) but were not implemented in the study design referring to the players of the study sample. In addition, only players that played the whole specific match were included. Since offensive players are substituted more frequently, this results in a smaller sample size for offensive positions (see Supplementary Table 4; Bradley et al., 2014). Because only starters are included, the results of the current study are not transferable to substitutes. Furthermore, the goal of this study was to describe the technical performance and to assess the typical technical requirements of players in the German Bundesliga. Analyzing the ratio of successful actions would add another level of evaluating and interpreting the results by assessing the quality of the respective technical actions. Future studies could focus on the success rate of actions and thus evaluate the quality of technical match performance. Moreover, the positions and tactical formations were observed at the beginning (first 15 min) of the respective match. Therefore, possible position and formation changes were not considered. However, the playing positions and formations indeed were reviewed by an experienced match analyst of a German Bundesliga team. In the future, these changes in tactical formations should be considered to obtain more precise and accurate results. To the best of the authors’ knowledge, no study has been published to date that investigated the frequency of formation changes of single teams during games. Further, to expand the gain of knowledge, studies should also consider substitutes. This could also potentially help to increase the sample size and, therefore, improve the robustness of the gained insights. In addition, dividing players that switched the playing position while tactical formation stayed constant and players that changed playing position and tactical formation could be profitable. Furthermore, the goalkeeper is becoming more and more important in modern build-up play. Future studies could also investigate the effect of tactical formation on the match performance of a goalkeeper.

## Conclusion

This study revealed that not only the playing position in a specific tactical formation, but also the individuality of the respective player influences the technical match performance of professional soccer players. Depending on the technical performance parameter, the positional role (i.e., playing position in a respective tactical formation) explains 3–44% of the variability due to the switch in playing position. The interindividual differences how players adapted or maintained their technical performance were large. Therefore, the manner (i.e., magnitude and direction of performance changes) in which the positional role influences the technical match performance depends on the individual player.

The findings of this study can help coaches interpret the technical match performance of single players after switching positional roles. Hence, it is worthwhile to adapt training programs not only to the positional role but also to the respective player. The results suggest that the size of the impact of tactical factors (i.e., positional role) is profoundly dependent on the individual player. When coaches have their players play in different positional roles, they need to consider not only the tactical position but also the individuality of each athlete. Further, scouts need to be aware of the extent of the influence of each the positional role and the individuality of the player when interpreting technical match performances of possible transfer candidates.

## Data Availability Statement

The data analyzed in this study is subject to the following licenses/restrictions: The authors thank the Deutsche Fußball Liga (DFL) for providing the match data used in this study. Further, there are no patents, products in development or marketed products associated with this research to declare. The data that support the findings of this study are available from the Deutsche Fußball Liga (DFL). Restrictions apply to the availability of these data, which were used under license for this study. The data is provided by a commercial company (Deltatre) and therefore the data is not freely available. Requests to access these datasets should be directed to Deutsche Fußball Liga (DFL), info@dfl.de.

## Ethics Statement

Ethical review and approval was not required for the study on human participants in accordance with the local legislation and institutional requirements. Written informed consent for participation was not required for this study in accordance with the national legislation and the institutional requirements.

## Author Contributions

LeoF, SA, TG, SH, and LeaF: conceptualization. LeoF and LeaF: data curation, investigation and visualization. LeoF, SA, and LeaF: formal analysis and software. AW, HW, and DJ: funding acquisition. LeoF, LeaF, SA, TG, DJ, HW, AW, and SH: methodology, writing—review and editing. LeoF, SA, and TG: project administration. AW and TG: resources. HW, DJ, AW, and SA: supervision. LeoF, SA, HW, DJ, AW, and SH: validation. LeoF: writing—original draft. All authors contributed to the article and approved the submitted version.

## Conflict of Interest

The authors have read the journal’s policy and have the following competing interests to declare: LeoF, TG, SH were employed by the commercial affiliation TSG 1899 Hoffenheim. SA was employed by the non-commercial limited liability company TSG ResearchLab gGmbH.

The remaining authors declare that the research was conducted in the absence of any commercial or financial relationships that could be construed as a potential conflict of interest.

## Publisher’s Note

All claims expressed in this article are solely those of the authors and do not necessarily represent those of their affiliated organizations, or those of the publisher, the editors and the reviewers. Any product that may be evaluated in this article, or claim that may be made by its manufacturer, is not guaranteed or endorsed by the publisher.
