# Supplementary material for: Does Technical Match Performance in Professional Soccer Depend on the Positional Role or the Individuality of the Player?
Source: Front Psychol. 2022 May 31;13:813206. doi: 10.3389/fpsyg.2022.813206 (PMC9197445; doi:10.3389/fpsyg.2022.813206)
Supplement: Supplementary file 1 [file Data_Sheet_1.docx]

**Supplementary Table 1.** Number of players per position (center back, full back, central midfielder, wide midfielder, forward) depending on the tactical formation.

|  | | playing position | | | | |
| --- | --- | --- | --- | --- | --- | --- |
|  |  | center back | full back | central midfielder | Wide midfielder | forward |
| tactical formation | 4-4-2 | 2 | 2 | 2 | 2 | 2 |
|  | 4-4-2 diamond | 2 | 2 | 2 | 2 | 2 |
|  | 4-2-2-2 | 2 | 2 | 2 | 2 | 2 |
|  | 4-3-3 | 2 | 2 | 3 | 2 | 1 |
|  | 4-5-1 | 2 | 2 | 3 | 2 | 1 |
|  | 4-2-3-1 | 2 | 2 | 3 | 2 | 1 |
|  | 3-4-3 | 3 | 2 | 2 | 2 | 1 |
|  | 3-5-2 | 3 | 2 | 3 | 0 | 2 |

**Supplementary Table 2.** Descriptive values (mean ±SD), t-test results, and ES of each player of the study sample for dribblings, short passes, medium passes, long passes, and ball possessions between playing positions.

|  | **Position 1** | **Position 2** | **t-value** | **df** | **p-value** | **ES** |
| --- | --- | --- | --- | --- | --- | --- |
| **Player 1** | **CM (4222)**  **[n=9]** | **CB (343)**  **[n=4]** |  |  |  |  |
| Dribblings | 0.11 ±0.31 | 0.00 ±0.00 | 0.65 | 11 | 0.53 | 0.28 |
| Short Passes | 8.56 ±5.04 | 12.00 ±2.35 | -1.20 | 11 | 0.26 | 1.66 |
| Medium Passes | 23.89 ±12.82 | 22.00 ±7.58 | 0.25 | 11 | 0.81 | 0.55 |
| Long Passes | 2.56 ±1.83 | 3.75 ±3.03 | -0.81 | 11 | 0.44 | 0.81 |
| Ball Possessions | 50.67 ±14.84 | 48.75 ±6.72 | 0.23 | 11 | 0.82 | 0.54 |
|  |  |  |  |  |  |  |
| **Player 2** | **CM (4231)**  **[n=5]** | **CB (451)**  **[n=6]** |  |  |  |  |
| Dribblings | 0.00+0.00 | 0.00 ±0.00 | 0.00 | 9 | >0.99 | 0.00 |
| Short Passes | 13.00 ±3.79 | 7.17 ±4.52 | 2.07 | 9 | 0.07 | 2.85 |
| Medium Passes | 25.40 ±7.39 | 33.67 ±16.19 | -0.95 | 9 | 0.37 | 2.36 |
| Long Passes | 1.60 ±1.02 | 3.50 ±2.06 | -1.70 | 9 | 0.12 | 1.50 |
| Ball Possessions | 57.40 ±10.19 | 58.33 ±19.11 | -0.89 | 9 | 0.93 | 0.24 |
|  |  |  |  |  |  |  |
| **Player 3** | **CB (4231)**  **[n=9]** | **FB (4231)**  **[n=6]** |  |  |  |  |
| Dribblings | 0.44 ±0.68 | 2.00 ±1.15 | -3.04 | 13 | <0.01 | 1.67 |
| Short Passes | 20.44 ±9.89 | 32.00 ±9.64 | -2.08 | 13 | 0.06 | 3.69 |
| Medium Passes | 40.33 ±14.77 | 27.50 ±11.79 | 1.66 | 13 | 0.12 | 3.47 |
| Long Passes | 4.78 ±2.20 | 2.17 ±1.07 | 2.52 | 13 | 0.03 | 1.97 |
| Ball Possessions | 79.11 ±17.60 | 90.17 ±21.08 | -1.02 | 13 | 0.33 | 2.54 |
|  |  |  |  |  |  |  |
| **Player 4** | **WM (451)**  **[n=4]** | **FB (352)**  **[n=4]** |  |  |  |  |
| Dribblings | 1.75 ±1.30 | 1.25 ±0.43 | 0.63 | 6 | 0.55 | 0.54 |
| Short Passes | 7.75 ±1.79 | 12.25 ±8.32 | -0.92 | 6 | 0.40 | 2.00 |
| Medium Passes | 9.50 ±4.27 | 10.75 ±5.31 | -0.32 | 6 | 0.76 | 0.57 |
| Long Passes | 1.75 ±1.79 | 3.25 ±0.83 | -1.32 | 6 | 0.24 | 1.31 |
| Ball Possessions | 37.00 ±4.18 | 48.50 ±11.28 | -1.66 | 6 | 0.15 | 4.14 |
|  |  |  |  |  |  |  |
| **Player 5** | **CM (442 diamond)**  **[n=7]** | **F (433)**  **[n=8]** |  |  |  |  |
| Dribblings | 2.14 ±1.46 | 0.75 ±0.97 | 2.05 | 13 | 0.06 | 1.27 |
| Short Passes | 24.71 ±4.59 | 18.38 ±3.46 | 2.83 | 13 | 0.01 | 3.18 |
| Medium Passes | 25.00 ±7.73 | 22.63 ±5.17 | 0.66 | 13 | 0.52 | 0.94 |
| Long Passes | 4.43 ±2.50 | 4.38 ±2.39 | 0.39 | 13 | 0.97 | 0.03 |
| Ball Possessions | 78.86 ±7.38 | 63.38 ±9.91 | 2.75 | 13 | 0.01 | 4.56 |
|  |  |  |  |  |  |  |
| **Player 6** | **WM (4222)**  **[n=8]** | **CM (352)**  **[n=6]** |  |  |  |  |
| Dribblings | 1.13 ±0.93 | 1.17 ±1.21 | -0.07 | 12 | 0.95 | 0.04 |
| Short Passes | 19.13 ±6.21 | 19.00 ±4.00 | 0.04 | 12 | 0.97 | 0.05 |
| Medium Passes | 15.63 ±7.16 | 18.00 ±7.87 | -0.55 | 12 | 0.60 | 0.87 |
| Long Passes | 3.38 ±2.50 | 3.17 ±2.19 | 0.15 | 12 | 0.88 | 0.14 |
| Ball Possessions | 57.63 ±5.27 | 57.67 ±6.52 | -0.01 | 12 | 0.99 | 0.02 |
|  |  |  |  |  |  |  |
| **Player 7** | **CM (442 diamond)**  **[n=6]** | **WM (442 diamond)**  **[n=10]** |  |  |  |  |
| Dribblings | 0.67 ±0.75 | 2.00 ±1.61 | -1.78 | 14 | 0.10 | 1.17 |
| Short Passes | 21.17 ±9.17 | 17.90 ±7.88 | 0.75 | 14 | 0.49 | 1.13 |
| Medium Passes | 20.83 ±10.25 | 12.10 ±5.26 | 2.10 | 14 | 0.05 | 3.29 |
| Long Passes | 4.50 ±3.20 | 1.70 ±1.00 | 2.40 | 14 | 0.03 | 2.09 |
| Ball Possessions | 63.50 ±18.73 | 49.80 ±10.57 | 1.75 | 14 | 0.10 | 3.73 |
|  |  |  |  |  |  |  |
| **Player 8** | **WM (442 diamond)**  **[n=7]** | **CM (433)**  **[n=12]** |  |  |  |  |
| Dribblings | 0.29 ±0.45 | 0.83 ±0.80 | -1.57 | 17 | 0.13 | 0.67 |
| Short Passes | 18.29 ±6.32 | 16.25 ±7.07 | 0.60 | 17 | 0.56 | 0.78 |
| Medium Passes | 20.86 ±6.75 | 21.17 ±9.55 | -0.07 | 17 | 0.94 | 0.11 |
| Long Passes | 2.29 ±1.83 | 1.50 ±1.61 | 0.92 | 17 | 0.37 | 0.61 |
| Ball Possessions | 57.29 ±11.35 | 55.17 ±18.68 | 0.26 | 17 | 0.80 | 0.53 |
|  |  |  |  |  |  |  |
| Player 9 | **WM (442 diamond)**  **[n=7]** | **CM (433)**  **[n=7]** |  |  |  |  |
| Dribblings | 0.86 ±0.64 | 0.43 ±0.49 | 1.30 | 12 | 0.22 | 0.57 |
| Short Passes | 22.14 ±6.20 | 15.14 ±2.80 | 2.52 | 12 | 0.03 | 3.30 |
| Medium Passes | 14.14 ±2.10 | 18.00 ±3.85 | -2.15 | 12 | 0.05 | 2.24 |
| Long Passes | 1.57 ±1.68 | 2.00 ±0.93 | -0.55 | 12 | 0.59 | 0.38 |
| Ball Possessions | 53.71 ±6.54 | 50.29 ±8.78 | 0.77 | 12 | 0.46 | 1.24 |
|  |  |  |  |  |  |  |
| Player 10 | **WM (442 diamond)**  **[n=4]** | **CM (433)**  **[n=5]** |  |  |  |  |
| Dribblings | 2.25 ±1.30 | 2.80 ±2.40 | -0.36 | 7 | 0.73 | 0.40 |
| Short Passes | 23.50 ±8.53 | 26.00 ±17.30 | -0.23 | 7 | 0.82 | 0.68 |
| Medium Passes | 18.25 ±6.68 | 21.00+5.76 | -0.58 | 7 | 0.58 | 1.11 |
| Long Passes | 4.00 ±2.12 | 4.20 ±3.66 | -0.08 | 7 | 0.93 | 0.12 |
| Ball Possessions | 71.50 ±13.39 | 77.20 ±15.65 | -0.51 | 7 | 0.63 | 1.49 |
|  |  |  |  |  |  |  |
| Player 11 | **WM (433)**  **[n=5]** | **CM (4231)**  **[n=11]** |  |  |  |  |
| Dribblings | 2.80 ±1.47 | 1.64 ±1.07 | 1.67 | 14 | 0.12 | 1.07 |
| Short Passes | 43.00 ±24.39 | 25.55 ±6.51 | 2.06 | 14 | 0.06 | 5.12 |
| Medium Passes | 24.80 ±7.88 | 17.82 ±8.99 | 1.40 | 14 | 0.18 | 2.37 |
| Long Passes | 1.60 ±1.02 | 2.09 ±1.38 | -0.67 | 14 | 0.52 | 0.43 |
| Ball Possessions | 88.60 ±28.95 | 63.00 ±10.00 | 2.44 | 14 | 0.03 | 6.52 |
|  |  |  |  |  |  |  |
| Player 12 | **WM (433)**  **[n=8]** | **F (352)**  **[n=5]** |  |  |  |  |
| Dribblings | 1.50 ±1.80 | 1.20 ±1.47 | 0.29 | 11 | 0.78 | 0.23 |
| Short Passes | 12.63 ±4.85 | 11.20 ±4.35 | 0.49 | 11 | 0.63 | 0.66 |
| Medium Passes | 10.50 ±4.53 | 7.00 ±2.28 | 1.48 | 11 | 0.17 | 1.82 |
| Long Passes | 0.25 ±0.66 | 0.20 ±0.40 | 0.14 | 11 | 0.89 | 0.07 |
| Ball Possessions | 36.50 ±10.07 | 32.80 ±4.45 | 0.71 | 11 | 0.49 | 1.31 |
|  |  |  |  |  |  |  |
| Player 13 | **F (433)**  **[n=9]** | **WM (4231)**  **[n=4]** |  |  |  |  |
| Dribblings | 0.78 ±1.03 | 1.75 ±1.79 | -1.14 | 11 | 0.28 | 0.87 |
| Short Passes | 11.33 ±4.27 | 15.50 ±7.53 | -1.16 | 11 | 0.27 | 1.83 |
| Medium Passes | 9.11 ±4.75 | 16.00 ±4.90 | -2.20 | 11 | 0.05 | 3.15 |
| Long Passes | 1.00 ±0.67 | 2.00 ±1.58 | -1.48 | 11 | 0.17 | 1.04 |
| Ball Possessions | 39.33 ±7.73 | 48.75 ±10.66 | -1.65 | 11 | 0.13 | 3.22 |
|  |  |  |  |  |  |  |

[CB = Center Back; FB = Full Back; CM = Central Midfielder; WM = Wide Midfielder; F = Forward]

**Supplementary Table 3.** Descriptive values (mean ± SD) per position (center back, full back, central midfielder, wide midfielder, forward) depending on the tactical formation (normative data).

| **Position** | **Formation** | **Sample** | **Mean** | **SD** | **Position** | | **Formation** | **Sample** | **Mean** | **SD** |  |
| --- | --- | --- | --- | --- | --- | --- | --- | --- | --- | --- | --- |
| **Ball Possessions** | | | | | | **Dribblings** | | | | | |
| center back | 4-4-2 | 32 | 64.81 | 18.48 | center back | | 4-4-2 | 32 | 0.31 | 0.54 |  |
|  | 4-4-2 diamond | 121 | 63.16 | 19.59 |  | | 4-4-2 diamond | 121 | 0.23 | 0.57 |  |
|  | 4-2-2-2 | 89 | 62.30 | 21.20 |  | | 4-2-2-2 | 89 | 0.20 | 0.48 |  |
|  | 4-3-3 | 209 | 80.54 | 27.28 |  | | 4-3-3 | 209 | 0.19 | 0.45 |  |
|  | 4-5-1 | 85 | 60.91 | 20.15 |  | | 4-5-1 | 85 | 0.09 | 0.40 |  |
|  | 4-2-3-1 | 195 | 73.88 | 24.21 |  | | 4-2-3-1 | 195 | 0.19 | 0.46 |  |
|  | 3-4-3 | 212 | 64.45 | 19.75 |  | | 3-4-3 | 212 | 0.23 | 0.56 |  |
|  | 3-5-2 | 184 | 59.86 | 18.24 |  | | 3-5-2 | 184 | 0.22 | 0.54 |  |
| full back | 4-4-2 | 29 | 58.62 | 13.44 | full back | | 4-4-2 | 29 | 0.79 | 0.94 |  |
|  | 4-4-2 diamond | 110 | 65.67 | 15.83 |  | | 4-4-2 diamond | 110 | 1.09 | 1.24 |  |
|  | 4-2-2-2 | 79 | 65.48 | 16.82 |  | | 4-2-2-2 | 79 | 0.90 | 1.10 |  |
|  | 4-3-3 | 183 | 72.81 | 19.49 |  | | 4-3-3 | 183 | 0.77 | 1.11 |  |
|  | 4-5-1 | 82 | 59.44 | 14.06 |  | | 4-5-1 | 82 | 0.80 | 1.06 |  |
|  | 4-2-3-1 | 181 | 74.67 | 19.39 |  | | 4-2-3-1 | 181 | 1.17 | 1.28 |  |
|  | 3-4-3 | 131 | 62.49 | 14.60 |  | | 3-4-3 | 131 | 1.38 | 1.45 |  |
|  | 3-5-2 | 118 | 58.69 | 13.33 |  | | 3-5-2 | 118 | 1.51 | 1.68 |  |
| central midfielder | 4-4-2 | 24 | 57.38 | 13.41 | central midfielder | | 4-4-2 | 24 | 0.33 | 0.64 |  |
|  | 4-4-2 diamond | 83 | 61.58 | 16.94 |  | | 4-4-2 diamond | 83 | 0.94 | 1.18 |  |
|  | 4-2-2-2 | 67 | 64.85 | 20.75 |  | | 4-2-2-2 | 67 | 0.61 | 1.09 |  |
|  | 4-3-3 | 221 | 64.18 | 22.84 |  | | 4-3-3 | 221 | 0.75 | 1.05 |  |
|  | 4-5-1 | 101 | 52.68 | 15.74 |  | | 4-5-1 | 101 | 0.84 | 1.23 |  |
|  | 4-2-3-1 | 210 | 66.23 | 21.72 |  | | 4-2-3-1 | 210 | 0.80 | 1.07 |  |
|  | 3-4-3 | 98 | 60.94 | 16.35 |  | | 3-4-3 | 98 | 0.65 | 0.90 |  |
|  | 3-5-2 | 123 | 54.74 | 14.27 |  | | 3-5-2 | 123 | 1.04 | 1.38 |  |
| wide mdifielder | 4-4-2 | 11 | 48.36 | 9.27 | wide mdifielder | | 4-4-2 | 11 | 2.45 | 1.81 |  |
|  | 4-4-2 diamond | 77 | 56.51 | 14.52 |  | | 4-4-2 diamond | 77 | 1.18 | 1.32 |  |
|  | 4-2-2-2 | 44 | 47.59 | 13.94 |  | | 4-2-2-2 | 44 | 2.36 | 2.28 |  |
|  | 4-3-3 | 88 | 51.91 | 17.97 |  | | 4-3-3 | 88 | 2.24 | 1.97 |  |
|  | 4-5-1 | 43 | 42.00 | 14.06 |  | | 4-5-1 | 43 | 2.07 | 1.89 |  |
|  | 4-2-3-1 | 112 | 53.32 | 15.98 |  | | 4-2-3-1 | 112 | 2.40 | 2.22 |  |
|  | 3-4-3 | 85 | 42.35 | 13.05 |  | | 3-4-3 | 85 | 1.47 | 1.74 |  |
| forward | 4-4-2 | 18 | 35.56 | 8.37 | forward | | 4-4-2 | 18 | 1.17 | 1.69 |  |
|  | 4-4-2 diamond | 57 | 37.75 | 8.60 |  | | 4-4-2 diamond | 57 | 1.75 | 1.98 |  |
|  | 4-2-2-2 | 48 | 38.81 | 12.23 |  | | 4-2-2-2 | 48 | 1.35 | 1.68 |  |
|  | 4-3-3 | 74 | 40.70 | 14.58 |  | | 4-3-3 | 74 | 0.54 | 0.83 |  |
|  | 4-5-1 | 22 | 31.32 | 7.29 |  | | 4-5-1 | 22 | 0.95 | 1.62 |  |
|  | 4-2-3-1 | 54 | 36.83 | 11.04 |  | | 4-2-3-1 | 54 | 1.22 | 1.19 |  |
|  | 3-4-3 | 43 | 46.09 | 12.93 |  | | 3-4-3 | 43 | 1.44 | 1.39 |  |
|  | 3-5-2 | 67 | 37.88 | 9.55 |  | | 3-5-2 | 67 | 1.51 | 1.53 |  |
| **Medium Passes** | | | | | | **Short Passes** | | | | | |
| center back | 4-4-2 | 32 | 33.47 | 14.33 | center back | | 4-4-2 | 32 | 8.22 | 4.80 |  |
|  | 4-4-2 diamond | 121 | 30.40 | 15.24 |  | | 4-4-2 diamond | 121 | 9.69 | 8.05 |  |
|  | 4-2-2-2 | 89 | 29.94 | 16.61 |  | | 4-2-2-2 | 89 | 10.57 | 8.03 |  |
|  | 4-3-3 | 209 | 45.95 | 21.05 |  | | 4-3-3 | 209 | 13.86 | 10.14 |  |
|  | 4-5-1 | 85 | 29.28 | 16.20 |  | | 4-5-1 | 85 | 10.14 | 6.25 |  |
|  | 4-2-3-1 | 195 | 38.67 | 18.20 |  | | 4-2-3-1 | 195 | 14.77 | 9.37 |  |
|  | 3-4-3 | 212 | 29.21 | 15.09 |  | | 3-4-3 | 212 | 13.57 | 9.33 |  |
|  | 3-5-2 | 184 | 24.67 | 13.58 |  | | 3-5-2 | 184 | 13.26 | 8.63 |  |
| full back | 4-4-2 | 29 | 17.90 | 6.07 | full back | | 4-4-2 | 29 | 8.62 | 4.46 |  |
|  | 4-4-2 diamond | 110 | 19.70 | 9.10 |  | | 4-4-2 diamond | 110 | 13.99 | 7.88 |  |
|  | 4-2-2-2 | 79 | 19.57 | 8.38 |  | | 4-2-2-2 | 79 | 14.63 | 8.66 |  |
|  | 4-3-3 | 183 | 25.14 | 11.49 |  | | 4-3-3 | 183 | 18.11 | 11.71 |  |
|  | 4-5-1 | 82 | 17.65 | 7.03 |  | | 4-5-1 | 82 | 12.65 | 7.62 |  |
|  | 4-2-3-1 | 181 | 22.85 | 10.58 |  | | 4-2-3-1 | 181 | 21.49 | 13.42 |  |
|  | 3-4-3 | 131 | 16.76 | 7.85 |  | | 3-4-3 | 131 | 14.29 | 7.57 |  |
|  | 3-5-2 | 118 | 15.69 | 6.80 |  | | 3-5-2 | 118 | 13.39 | 6.16 |  |
| central midfielder | 4-4-2 | 24 | 24.25 | 9.62 | central midfielder | | 4-4-2 | 24 | 15.42 | 6.57 |  |
|  | 4-4-2 diamond | 83 | 21.12 | 9.33 |  | | 4-4-2 diamond | 83 | 18.17 | 10.69 |  |
|  | 4-2-2-2 | 67 | 24.34 | 11.43 |  | | 4-2-2-2 | 67 | 19.37 | 12.53 |  |
|  | 4-3-3 | 221 | 25.10 | 13.10 |  | | 4-3-3 | 221 | 19.80 | 12.04 |  |
|  | 4-5-1 | 101 | 18.65 | 8.66 |  | | 4-5-1 | 101 | 15.30 | 8.69 |  |
|  | 4-2-3-1 | 210 | 24.13 | 12.95 |  | | 4-2-3-1 | 210 | 23.32 | 12.70 |  |
|  | 3-4-3 | 98 | 21.10 | 9.31 |  | | 3-4-3 | 98 | 20.00 | 9.96 |  |
|  | 3-5-2 | 123 | 17.73 | 8.41 |  | | 3-5-2 | 123 | 16.49 | 7.56 |  |
| wide mdifielder | 4-4-2 | 11 | 14.91 | 3.65 | wide mdifielder | | 4-4-2 | 11 | 11.73 | 5.87 |  |
|  | 4-4-2 diamond | 77 | 17.78 | 7.83 |  | | 4-4-2 diamond | 77 | 18.70 | 9.75 |  |
|  | 4-2-2-2 | 44 | 12.64 | 6.68 |  | | 4-2-2-2 | 44 | 14.36 | 7.97 |  |
|  | 4-3-3 | 88 | 14.47 | 6.80 |  | | 4-3-3 | 88 | 16.63 | 11.06 |  |
|  | 4-5-1 | 43 | 10.30 | 4.45 |  | | 4-5-1 | 43 | 11.49 | 8.04 |  |
|  | 4-2-3-1 | 112 | 14.29 | 6.53 |  | | 4-2-3-1 | 112 | 18.11 | 10.93 |  |
|  | 3-4-3 | 85 | 9.05 | 6.17 |  | | 3-4-3 | 85 | 13.96 | 7.64 |  |
| forward | 4-4-2 | 18 | 10.22 | 4.25 | forward | | 4-4-2 | 18 | 10.29 | 5.73 |  |
|  | 4-4-2 diamond | 57 | 8.28 | 5.00 |  | | 4-4-2 diamond | 57 | 14.01 | 9.38 |  |
|  | 4-2-2-2 | 48 | 9.25 | 6.33 |  | | 4-2-2-2 | 48 | 14.14 | 9.44 |  |
|  | 4-3-3 | 74 | 10.58 | 7.65 |  | | 4-3-3 | 74 | 16.75 | 11.16 |  |
|  | 4-5-1 | 22 | 6.05 | 2.84 |  | | 4-5-1 | 22 | 12.43 | 7.76 |  |
|  | 4-2-3-1 | 54 | 8.15 | 3.70 |  | | 4-2-3-1 | 54 | 19.07 | 12.15 |  |
|  | 3-4-3 | 43 | 11.26 | 7.99 |  | | 3-4-3 | 43 | 15.09 | 8.95 |  |
|  | 3-5-2 | 67 | 7.96 | 3.70 |  | | 3-5-2 | 67 | 13.93 | 7.54 |  |
| **Long Passes** | | | | | |  |  |  |  |  |  |
| center back | 4-4-2 | 32 | 5.53 | 2.96 |  |  |  |  |  |  |  |
|  | 4-4-2 diamond | 121 | 6.13 | 4.42 |  |  |  |  |  |  |  |
|  | 4-2-2-2 | 89 | 5.54 | 3.66 |  |  |  |  |  |  |  |
|  | 4-3-3 | 209 | 6.02 | 4.39 |  |  |  |  |  |  |  |
|  | 4-5-1 | 85 | 5.55 | 3.36 |  |  |  |  |  |  |  |
|  | 4-2-3-1 | 195 | 5.36 | 3.69 |  |  |  |  |  |  |  |
|  | 3-4-3 | 212 | 5.46 | 3.77 |  |  |  |  |  |  |  |
|  | 3-5-2 | 184 | 4.77 | 3.19 |  |  |  |  |  |  |  |
| full back | 4-4-2 | 29 | 4.14 | 2.66 |  |  |  |  |  |  |  |
|  | 4-4-2 diamond | 110 | 3.67 | 2.54 |  |  |  |  |  |  |  |
|  | 4-2-2-2 | 79 | 3.70 | 2.75 |  |  |  |  |  |  |  |
|  | 4-3-3 | 183 | 3.24 | 2.53 |  |  |  |  |  |  |  |
|  | 4-5-1 | 82 | 3.87 | 2.56 |  |  |  |  |  |  |  |
|  | 4-2-3-1 | 181 | 3.06 | 2.65 |  |  |  |  |  |  |  |
|  | 3-4-3 | 131 | 3.08 | 2.28 |  |  |  |  |  |  |  |
|  | 3-5-2 | 118 | 2.66 | 2.30 |  |  |  |  |  |  |  |
| central midfielder | 4-4-2 | 24 | 2.08 | 1.59 |  |  |  |  |  |  |  |
|  | 4-4-2 diamond | 83 | 3.35 | 3.21 |  |  |  |  |  |  |  |
|  | 4-2-2-2 | 67 | 3.28 | 2.71 |  |  |  |  |  |  |  |
|  | 4-3-3 | 221 | 3.19 | 2.78 |  |  |  |  |  |  |  |
|  | 4-5-1 | 101 | 2.55 | 2.11 |  |  |  |  |  |  |  |
|  | 4-2-3-1 | 210 | 2.67 | 2.74 |  |  |  |  |  |  |  |
|  | 3-4-3 | 98 | 2.22 | 1.80 |  |  |  |  |  |  |  |
|  | 3-5-2 | 123 | 2.37 | 2.11 |  |  |  |  |  |  |  |
| wide mdifielder | 4-4-2 | 11 | 1.36 | 1.36 |  |  |  |  |  |  |  |
|  | 4-4-2 diamond | 77 | 2.56 | 2.16 |  |  |  |  |  |  |  |
|  | 4-2-2-2 | 44 | 1.68 | 1.88 |  |  |  |  |  |  |  |
|  | 4-3-3 | 88 | 1.35 | 1.47 |  |  |  |  |  |  |  |
|  | 4-5-1 | 43 | 1.26 | 1.38 |  |  |  |  |  |  |  |
|  | 4-2-3-1 | 112 | 1.33 | 1.42 |  |  |  |  |  |  |  |
|  | 3-4-3 | 85 | 1.06 | 1.37 |  |  |  |  |  |  |  |
| forward | 4-4-2 | 18 | 0.56 | 0.86 |  |  |  |  |  |  |  |
|  | 4-4-2 diamond | 57 | 0.53 | 0.85 |  |  |  |  |  |  |  |
|  | 4-2-2-2 | 48 | 0.75 | 1.49 |  |  |  |  |  |  |  |
|  | 4-3-3 | 74 | 1.01 | 1.65 |  |  |  |  |  |  |  |
|  | 4-5-1 | 22 | 0.36 | 0.73 |  |  |  |  |  |  |  |
|  | 4-2-3-1 | 54 | 0.50 | 0.75 |  |  |  |  |  |  |  |
|  | 3-4-3 | 43 | 1.09 | 1.25 |  |  |  |  |  |  |  |
|  | 3-5-2 | 67 | 0.57 | 0.82 |  |  |  |  |  |  |  |

**Supplementary Table 4.** Descriptive values (mean ± SD) depending on the playing position (normative data).

| **position** | **sample** | **Mean** | **SD** | **position** | **sample** | **Mean** | **SD** |
| --- | --- | --- | --- | --- | --- | --- | --- |
| **Ball Possessions** | | | | **Dribblings** | | | |
| center back | 1127 | 67.75 | 23.21 | center back | 1127 | 0.20 | 0.50 |
| full back | 913 | 66.73 | 17.85 | full back | 913 | 1.09 | 1.31 |
| central midfielder | 927 | 61.44 | 19.88 | central midfielder | 927 | 0.80 | 1.12 |
| wide midfielder | 460 | 49.83 | 15.97 | wide midfielder | 460 | 1.96 | 1.97 |
| forward | 383 | 38.81 | 11.76 | forward | 383 | 1.24 | 1.51 |
| **Medium Passes** | | | | **Short Passes** | | | |
| center back | 1127 | 33.52 | 18.33 | center back | 1127 | 12.72 | 9.04 |
| full back | 913 | 20.22 | 9.80 | full back | 913 | 16.03 | 10.35 |
| central midfielder | 927 | 22.35 | 11.49 | central midfielder | 927 | 19.40 | 11.24 |
| wide midfielder | 460 | 13.42 | 7.12 | wide midfielder | 460 | 16.03 | 9.84 |
| forward | 383 | 9.07 | 5.86 | forward | 383 | 12.14 | 6.09 |
| **Long Passes** | | | |  |  |  |  |
| center back | 1127 | 5.52 | 3.82 |  |  |  |  |
| full back | 913 | 3.28 | 2.54 |  |  |  |  |
| central midfielder | 927 | 2.79 | 2.57 |  |  |  |  |
| wide midfielder | 460 | 1.52 | 1.67 |  |  |  |  |
| forward | 383 | 0.71 | 1.18 |  |  |  |  |

**Supplementary Table 5.** Descriptive values (mean ± SD) depending on the tactical formation (normative data).

| **formation** | **sample** | **Mean** | **SD** | **Formation** | **sample** | **Mean** | **SD** |
| --- | --- | --- | --- | --- | --- | --- | --- |
| **Ball Possessions** | | | | **Dribblings** | | | |
| 4-4-2 | 114 | 55.46 | 17.01 | 4-4-2 | 114 | 0.78 | 1.22 |
| 4-4-2 diamond | 448 | 59.11 | 18.37 | 4-4-2 diamond | 448 | 0.93 | 1.32 |
| 4-2-2-2 | 327 | 58.17 | 20.50 | 4-2-2-2 | 327 | 0.91 | 1.47 |
| 4-3-3 | 775 | 66.99 | 25.41 | 4-3-3 | 775 | 0.75 | 1.22 |
| 4-5-1 | 333 | 53.65 | 18.07 | 4-5-1 | 333 | 0.81 | 1.31 |
| 4-2-3-1 | 752 | 66.21 | 23.21 | 4-2-3-1 | 752 | 1.00 | 1.44 |
| 3-4-3 | 569 | 58.70 | 18.58 | 3-4-3 | 569 | 0.85 | 1.27 |
| 3-5-2 | 492 | 55.31 | 16.77 | 3-5-2 | 492 | 0.91 | 1.38 |
| **Medium Passes** | | | | **Short Passes** | | | |
| 4-4-2 | 114 | 22.11 | 12.56 | 4-4-2 | 114 | 10.29 | 5.73 |
| 4-4-2 diamond | 448 | 21.07 | 12.60 | 4-4-2 diamond | 448 | 14.01 | 9.38 |
| 4-2-2-2 | 327 | 20.92 | 13.56 | 4-2-2-2 | 327 | 14.14 | 9.44 |
| 4-3-3 | 775 | 28.14 | 18.73 | 4-3-3 | 775 | 16.75 | 11.16 |
| 4-5-1 | 333 | 19.21 | 12.35 | 4-5-1 | 333 | 12.43 | 7.76 |
| 4-2-3-1 | 752 | 24.98 | 15.93 | 4-2-3-1 | 752 | 19.07 | 12.15 |
| 3-4-3 | 569 | 20.58 | 13.47 | 3-4-3 | 569 | 15.09 | 8.95 |
| 3-5-2 | 492 | 18.51 | 11.43 | 3-5-2 | 492 | 13.93 | 7.54 |
| **Long Passes** | | | |  |  |  |  |
| 4-4-2 | 114 | 3.26 | 2.90 |  |  |  |  |
| 4-4-2 diamond | 448 | 3.69 | 3.57 |  |  |  |  |
| 4-2-2-2 | 327 | 3.41 | 3.22 |  |  |  |  |
| 4-3-3 | 775 | 3.55 | 3.50 |  |  |  |  |
| 4-5-1 | 333 | 3.33 | 2.96 |  |  |  |  |
| 4-2-3-1 | 752 | 3.11 | 3.15 |  |  |  |  |
| 3-4-3 | 569 | 3.37 | 3.24 |  |  |  |  |
| 3-5-2 | 492 | 3.09 | 2.89 |  |  |  |  |

**Supplementary Table 6.** Data of contextual factors are presented as mean values ± SD.

| **formation** | **games** | **mean** | **SD** |
| --- | --- | --- | --- |
| **own team ranking (end of the season)** | | | |
| 4-4-2 | 16 | 13.50 | 2.48 |
| 4-4-2 dia. | 63 | 9.70 | 3.99 |
| 4-2-2-2 | 46 | 10.50 | 5.72 |
| 4-3-3 | 109 | 6.38 | 4.50 |
| 4-5-1 | 46 | 13.43 | 4.01 |
| 4-2-3-1 | 106 | 7.53 | 5.63 |
| 3-4-3 | 78 | 11.12 | 4.16 |
| 3-5-2 | 69 | 10.55 | 4.37 |
| **opposition team ranking (end of the season)** | | | |
| 4-4-2 | 16 | 8.44 | 5.27 |
| 4-4-2 dia. | 63 | 9.70 | 4.78 |
| 4-2-2-2 | 46 | 10.67 | 4.94 |
| 4-3-3 | 109 | 9.71 | 5.09 |
| 4-5-1 | 46 | 7.70 | 5.41 |
| 4-2-3-1 | 106 | 9.86 | 5.09 |
| 3-4-3 | 78 | 9.55 | 5.15 |
| 3-5-2 | 69 | 8.83 | 5.68 |
| **net game time [min]** | | | |
| 4-4-2 | 16 | 58.91 | 4.38 |
| 4-4-2 dia. | 63 | 56.23 | 3.94 |
| 4-2-2-2 | 46 | 56.98 | 4.19 |
| 4-3-3 | 109 | 58.73 | 4.25 |
| 4-5-1 | 46 | 57.84 | 3.90 |
| 4-2-3-1 | 106 | 58.30 | 4.65 |
| 3-4-3 | 78 | 56.46 | 4.00 |
| 3-5-2 | 69 | 56.32 | 3.91 |
| **points per game [quantity]** | | | |
| 4-4-2 | 16 | 1.00 | 1.26 |
| 4-4-2 dia. | 63 | 1.71 | 1.33 |
| 4-2-2-2 | 46 | 1.67 | 1.38 |
| 4-3-3 | 109 | 1.51 | 1.33 |
| 4-5-1 | 46 | 0.87 | 1.20 |
| 4-2-3-1 | 106 | 1.68 | 1.35 |
| 3-4-3 | 78 | 0.97 | 1.23 |
| 3-5-2 | 69 | 1.17 | 1.21 |
| **ball-possession [%]** | | | |
| 4-4-2 | 16 | 45.55 | 6.37 |
| 4-4-2 dia. | 63 | 50.05 | 7.35 |
| 4-2-2-2 | 46 | 48.09 | 8.17 |
| 4-3-3 | 109 | 53.92 | 9.13 |
| 4-5-1 | 46 | 44.32 | 8.32 |
| 4-2-3-1 | 106 | 51.98 | 8.99 |
| 3-4-3 | 78 | 50.09 | 8.01 |
| 3-5-2 | 69 | 46.63 | 7.65 |
| **venue (home [1] / away [2])** | | | |
| 4-4-2 | 16 | 1.50 | 0.52 |
| 4-4-2 dia. | 63 | 1.49 | 0.50 |
| 4-2-2-2 | 46 | 1.50 | 0.51 |
| 4-3-3 | 109 | 1.50 | 0.50 |
| 4-5-1 | 46 | 1.46 | 0.50 |
| 4-2-3-1 | 106 | 1.52 | 0.50 |
| 3-4-3 | 78 | 1.50 | 0.50 |
| 3-5-2 | 69 | 1.51 | 0.50 |

dia. = diamond

**Supplementary Table 7.** Contextual information about the players of the study sample.

| **Player** | **League games played** | **Nationality** | **Team (table position end of the season)** | **Involved in international competition** |
| --- | --- | --- | --- | --- |
| Player 1 | 24 | Germany | SC Freiburg [13] | No |
| Player 2 | 30 | Germany | FC Augsburg [15] | No |
| Player 3 | 28 | Senegal | Borussia Dortmund [2] | Champions League |
| Player 4 | 18 | Germany | Fortuna Düsseldorf [10] | No |
| Player 5 | 32 | Germany | SV Werder Bremen [8] | No |
| Player 6 | 30 | Austria | RB Leipzig [3] | Europa League |
| Player 7 | 31 | Ivory Coast | FSV Mainz 05 [12] | No |
| Player 8 | 24 | Germany | SV Werder Bremen [8] | No |
| Player 9 | 33 | Netherlands | SV Werder Bremen [8] | No |
| Player 10 | 26 | Germany | TSG 1899 Hoffenheim [9] | Champions League |
| Player 11 | 34 | Germany | Bayer 04 Leverkusen [4] | Europa League |
| Player 12 | 34 | France | Borussia Mönchengladbach [5] | No |
| Player 13 | 34 | Germany | Bayer 04 Leverkusen [4] | Europa League |
